# Quantification of the mobility potential of antibiotic resistance genes through multiplexed ddPCR linkage analysis

**DOI:** 10.1093/femsec/fiad031

**Published:** 2023-03-20

**Authors:** Magali de la Cruz Barron, David Kneis, Alan Xavier Elena, Kenyum Bagra, Thomas U Berendonk, Uli Klümper

**Affiliations:** Institute of Hydrobiology, Technische Universität Dresden, Dresden 01062, Zellescher Weg 40, Germany; Institute of Hydrobiology, Technische Universität Dresden, Dresden 01062, Zellescher Weg 40, Germany; Institute of Hydrobiology, Technische Universität Dresden, Dresden 01062, Zellescher Weg 40, Germany; Institute of Hydrobiology, Technische Universität Dresden, Dresden 01062, Zellescher Weg 40, Germany; Department of Civil Engineering, Indian Institute of Technology, Roorkee, Uttarakhand 247667, India; Institute of Hydrobiology, Technische Universität Dresden, Dresden 01062, Zellescher Weg 40, Germany; Institute of Hydrobiology, Technische Universität Dresden, Dresden 01062, Zellescher Weg 40, Germany

**Keywords:** antibiotic resistance, droplet digital PCR, mobile genetic elements, mobility, risk assessment

## Abstract

There is a clear need for global monitoring initiatives to evaluate the risks of antibiotic resistance genes (ARGs) towards human health. Therefore, not only ARG abundances within a given environment, but also their potential mobility, hence their ability to spread to human pathogenic bacteria needs to be quantified. We developed a novel, sequencing-independent method for assessing the linkage of an ARG to a mobile genetic element by statistical analysis of multiplexed droplet digital PCR (ddPCR) carried out on environmental DNA sheared into defined, short fragments. This allows quantifying the physical linkage between specific ARGs and mobile genetic elements, here demonstrated for the sulfonamide ARG *sul1* and the Class 1 integron integrase gene *intI1*. The method's efficiency is demonstrated using mixtures of model DNA fragments with either linked and unlinked target genes: Linkage of the two target genes can be accurately quantified based on high correlation coefficients between observed and expected values (R^2^) as well as low mean absolute errors (MAE) for both target genes, *sul1* (R^2^ = 0.9997, MAE = 0.71%, n = 24) and *intI1* (R^2^ = 0.9991, MAE = 1.14%, n = 24). Furthermore, we demonstrate that adjusting the fragmentation length of DNA during shearing allows controlling rates of false positives and false negative detection of linkage. The presented method allows rapidly obtaining reliable results in a labor- and cost-efficient manner.

## Teaser

A novel, rapid, and cost-efficient method to quantify the risk of environmental antibiotic resistance genes to end up in human pathogens has been developed.

## Introduction

The rise in antibiotic resistance in bacterial pathogens over time represents one of the biggest challenges in treating bacterial infections (Laxminarayan et al. 2013). Horizontal gene transfer, facilitated by mobile genetic elements (MGEs), has contributed significantly to the spread of antibiotic resistance genes (ARGs) among different hosts, accelerating this global health problem (Berendonk et al. 2015, Klümper et al. 2015). Addressing the problems associated with antibiotic resistance, requires action across the human, animal and environmental spheres within a ‘One Health’ concept (Queenan et al. 2016). Within this context, there is a clear need for global monitoring and risk assessment initiatives to assess the large-scale dissemination of antibiotic resistant bacteria and their associated ARGs across human, animal, but also the environmental spheres (Hernando-Amado et al. 2019). In the latter, this proves especially difficult due to the high complexity of gene transfer and the diversity of non-culturable microbes (Smalla et al. 2018, Huijbers et al. 2019).

The occurrence and distribution of antimicrobial resistant bacteria (ARB), ARGs and MGEs in different environments has been extensively documented using diverse approaches ranging from selective cultivation, qPCR of ARGs and mobility related genes and metagenomic sequencing (Barrón et al. 2018, Cacace et al. 2019, Pärnänen et al. 2019, Kampouris et al. 2021). However, the usual report of absolute and relative abundance of ARGs is not enough to accurately assess the risks of these markers on human health, as ARGs can be found in a variety of different hosts and on diverse mobile genetic elements. The genetic and host context in which these genes appear can result in varying risk levels.

Therefore, the urgent need for a conclusive framework addressing this issue in environmental samples is beyond dispute (Berendonk et al. 2015, Smalla et al. 2018, Huijbers et al. 2019). Recently, Zhang et al. (2021) proposed four key indicators to evaluate the health risk of environmental ARGs: (i) Human accessibility: Is the ARG shared between human and environmental microbiomes?; (ii) Human pathogenicity: Has the ARG been found in human pathogens?; (iii) Clinical relevance: Has the presence of the ARG been associated to clinical complications/worsened clinical outcome?; (iv) Mobility: Is the ARG encoded on a MGE that is implicated with the potential of allowing ARG transfer among bacteria, hence increasing the risk of an ARG being transferred to a pathogen? The first three criteria are reasonably easy to assess with the currently available data and methods. However, evaluating the linkage of these ARGs to MGEs, hence their mobility in environmental communities, is crucial but remains a challenge. Consequently, methods for quantitatively assessing the potential mobility of ARGs that are easily applicable and of low costs are urgently needed to include them in future global, environmental AMR monitoring frameworks. Here, such a quantitative method has been developed by taking advantage of multiplexed droplet digital PCR (ddPCR) technology.

The ddPCR technology is a binary endpoint measurement based on the random distribution of nucleic acid molecules extracted from a sample partitioned into thousands of volumetrically defined droplets within a water–oil emulsion with PCR reactions taking place in each individual droplet (Hindson et al. 2011). Using a fluorescent probe based approach with different fluorochromes for different target genes allows multiplexing their detection within the entire sample, but also within individual droplets (Whale et al. 2016). Since individual DNA molecules are randomly encapsulated within the droplets based on the Poisson distribution (Regan et al. 2015), the physical linkage between a target ARG and a target MGE on this DNA molecule can be detected and estimated by ddPCR linkage analysis using multiplexed assays in combination with statistical analysis.

Here, we developed a new duplex ddPCR approach to quantify the mobility potential of an ARG. The mobility potential is hereby infered as the percentage of this ARGs detected copies in direct physical linkage with a marker gene associated with a MGEs. This is here presented for the sulfonamide resistance gene *sul1* and its occurrence as a component of mobile Class 1 integron cassettes, assessed through its linkage with the integrase gene *intI1*. Although these two genes can occur individually in different environments and bacteria, they have been regularly described to co-occur in mobile integron cassettes in a wide variety of bacteria and environments (Gillings et al. 2015), and hence provide a perfect study system to demonstrate the validity of the proposed method.

To prove that multiplexed ddPCR can be used as a suitable, rapid, and less expensive approach to determine the linkage between ARGs and MGEs, model DNA mixtures with linkages ranging from 100% to 0% were analyzed. The validity of the linkage detection for each of the two genes was then assessed by comparing the linkage determined for each of the mixtures using the statistical evaluation of the duplex ddPCR assay with the theoretical, expected linkage percentage. To avoid a biased linkage detection when the two target genes occur individually (outside of an individual integron cassette) on the same chromosome, extracted DNA needs, prior to analysis, be sheared into short fragments. Based on the statistical probability evaluation of false positive detections, a shearing size of around 20 000 base pairs (20 kbp), approximately five to ten times the size of an average Class 1 integron cassette (Partridge et al. 2009), is suggested for optimal detection of the association of the ARG with Class 1 integrons. Using the presented multiplexed ddPCR approach we demonstrate its suitability by simultaneously assessing the abundance of the individual targets and their linkages in DNA from environmental samples from the influent and effluent of a wastewater treatment plant.

## Material and methods

### Linked and unlinked target DNA fragments

To prove the concept and optimize the methodology for determining the physical linkage between an ARG and a mobility marker using a ddPCR multiplexed protocol the sulfonamide resistance genes *sul1* and the Class 1 integron integrase gene *intI1* were selected. To analyze, if the linkage of these two genes can be correctly predicted using the ddPCR protocol, we used model DNA molecules on which either one of the two targets, or both targets linked on the same molecule, are present.

The pNORM plasmid (http://www.norman-network.net/) served as the linked control, as it contains a single copy of both target genes in close proximity of 250 bp (Cacace et al. 2019). Plasmid DNA was extracted from the *E. coli* host strain using the Monarch® Plasmid Miniprep Kit (NEB, Ipswich, MA, USA), linearized with restriction enzyme *BamHI* (Promega, Madison, WI, USA), and thereafter purified with the QIAquick PCR Purification Kit (Qiagen, Hilden, Germany), according to the protocols supplied by the manufacturers.

Purified PCR products of the *sul1* and *intI1* gene were used as the no linkage controls. PCR was carried out individually for each gene using GoTaq® Green Master Mix (Promega), with primers listed in Table 1, and linearized pNORM DNA as the template. The PCR program was set as follows: 95°C for 10 min followed by 35 cycles at 94°C for 30 s, 60°C for 30 s, 72°C for 30 s, and a final elongation cycle at 72°C for 10 min. Successful amplification and correct size of the *sul1* and *intI1* PCR products were confirmed by 1.5% agarose gel electrophoresis (40 min, 110 V) and PCR products were then purified with the QIAquick PCR Purification Kit (Qiagen) according to manufacturer's instructions.

**Table 1.** Primers and probes used in this study.

Name	Sequence	Target gene	Product size (bp)	Reference
**intI1LC5**	GATCGGTCGAATGCGTGT	*intI1* (class 1 integron integrase)		
**intI1LC1**	GCCTTGATGTTACCCGAGAG		196	(Barraud et al. 2010)
intI1-probe*	[HEX]ATTCCTGGCCGTGGTTCTGGG TTTT [BHQ1]			
**sul1-FW**	CGCACCGGAAACATCGCTGCAC	*sul1* (sulfonamide resistance dihydropteroate synthase)		
**sul1-RV**	TGAAGTTCCGCCGCAAGGCTCG		162	(Rocha et al. 2019)
**sul1-probe**	[FAM]CGCCACCGTTGGCCTTCCTGTAAA GGATCTGG [BBQ650]			This study

*Fluorophore and quencher differ from the reference.

Linearized pNORM with both targets linked as well as a 1 : 1 mixture of the individual, unlinked PCR products were prepared in DNAse free H_2_O (Qiagen) and adjusted to 1 ng µL^−1^. These two solutions were then used in mixtures of varying ratios to assess the detection of different linkage percentages (see below) using the ddPCR duplex detection protocol.

### ddPCR duplex detection protocol

To allow the simultaneous detection of two genes within one ddPCR assay, individual probe based PCR assays for each of the genes consisting of forward and reverse primer and a probe labeled with non-interfering fluorochromes are necessary. While for the *intI1* gene such an assay already existed (Barraud et al. 2010), for *sul1* an existing and well-validated qPCR primer set was used (Rocha et al. 2019). The matching fluorescent probe was designed for ddPCR detection of the *sul1* gene (Table 1). No cross-reactivity between the assays was detected when multiplexing the assays with the *intI1* probe labeled with HEX and the *sul1* probe labeled with FAM reporter fluorophores.

To quantify the two target genes *sul1* and *intI1*, we performed a duplexed ddPCR assays using the primers and probes listed in Table 1, and the QX200 Droplet Digital PCR System (Bio-Rad, Hercules, CA, USA). Reactions were prepared in 20 µL volume, containing: ddPCR Supermix for Probes (No dUTP, Bio-Rad), each of the four primers, and two probes (Table 1) at final concentrations of 900 nm for primers and 250 nM for probes, and 1 ng of DNA. This DNA could either be a mixture of the linked and non-linked targets created previously, or genomic DNA from sheared or non-sheared samples (see below).

Droplets were generated on a QX200 droplet generator (Bio-Rad), and transferred into a 96-well PCR plate (heat-sealed with a foil plate seal, Bio-Rad). PCR was carried out in a C1000 thermal cycler (Bio-Rad) using the following cycling conditions: 95°C for 10 min followed by 45 cycles at 94°C for 30 s, and 60°C for 90 s, and a final cycle at 98°C for 10 min. For higher fluorescence values of the positive droplets, resulting in a better droplet separation, 45 cycles were required and the ramp rate was set to 2.0°C/s. The droplets were read using a QX200 droplet reader and data was analyzed using QuantaSoft Software v1.4.0.99 (Bio-Rad).

### Calculation of the linkage of genes

**Figure 1.**
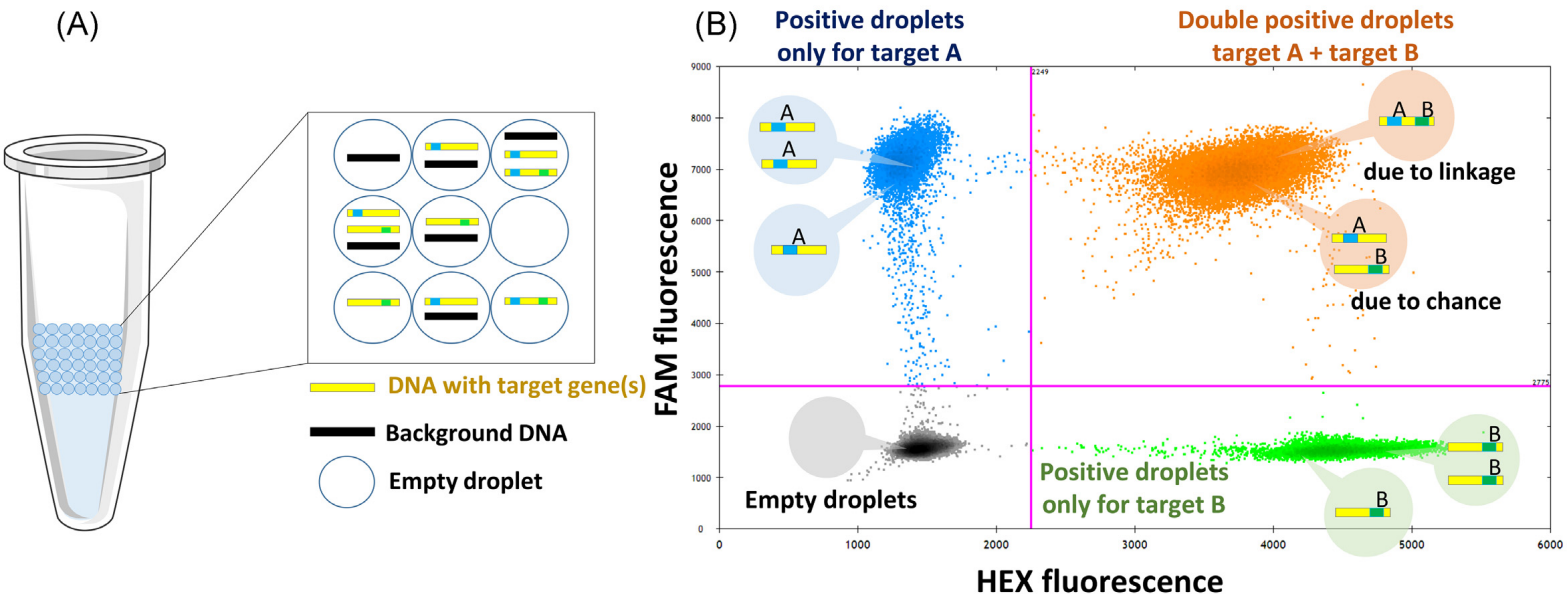
(**A**) Partitioning of PCR reaction into ca. 20 000 droplets of uniform size and volume, containing target and background DNA. (**B**) Data output from multiplex ddPCR experiments. Droplets form four clusters arranged orthogonally to each other. In grey: Empty droplets, negative for both targets (− −); Blue: positive droplets only for target A (+ −); Green: positive droplets only for target B (− +) and Orange: double-positive droplets (+ +) due to chance (no linkage) or due to physical linkage.

During the ddPCR partitioning process, DNA fragments are randomly Poisson distributed into thousands of droplets (∼20 000 per ddPCR assay) (Hindson et al. 2011). This means that individual droplets could either be empty or contain one or more DNA fragments with or without targets of interest (Fig. 1A). In each droplet the PCR reaction is performed and the fluorescence detected. After detection and data analysis, in multiplex ddPCR experiments four orthogonal clusters should be observed (Fig. 1B). Each droplet could either contain no target, only target A (*sul1*), only target B (*intI1*), or both targets either by chance (co-localization) or due to physical linkage on the same DNA fragment (Fig 1B).

Total concentrations of either target A or target B can simply be calculated based on the Poisson distribution and the amount of droplets positive for the specific target. The total number of physically linked molecules can then be calculated by determining the excess of double-positive droplets over that expected due to chance. To determine this linkage between the two target genes mathematically a modified version of the equations described by Regan et al. (Regan et al. 2015) for eukaryotic chromosomal linkage analysis needs to be applied:

If the two markers A and B are unlinked, their presence in individual droplets follows the Poisson distribution. Consequently, they will be partitioned according to the following equation, with N as the number of droplets analyzed from a ddPCR assay, N_A_ and N_B_ the number of droplets for the single target, N_E_ the number of empty, double-negative droplets and N_AB_ the number of double positive droplets:


(1) \begin{eqnarray*} {N}_A \times \ {N}_B = {N}_E\ \times {N}_{AB} \end{eqnarray*}

Rearranging this equation allows calculating the amount of double-positive droplets that appear purely by chance N_ch_ of both unlinked targets appearing in the same droplet:


(2) \begin{eqnarray*} {N}_{ch} = \frac{{{N}_A \times {N}_B}}{{{N}_E}}\ \end{eqnarray*}

When target A and B are physically linked (AB) additional double-positive droplets that are not based on chance appear. The amount of droplets that do not contain these linked targets N_notAB_ can now be calculated as the sum of empty, single-positive and chance-based double-positive droplets.


(3) \begin{eqnarray*} {N}_{not\underline {AB} } = {N}_E\ + {N}_A + {N}_B + \frac{{{N}_A \times {N}_B}}{{{N}_E}} \end{eqnarray*}

By resolving the Poisson distribution through the natural logarithm and subtracting N_notAB_ from the total number of measured droplets N_tot_ we calculate the concentration (average copies per droplet) of DNA molecules with linked targets λ_AB_ using:


(4) \begin{eqnarray*} {{\rm{\lambda }}}_{\underline {AB} } = \ln \left( {{N}_{tot}} \right)\ - {\rm{ln}}\left( {{N}_E + {N}_A + {N}_B + \frac{{{N}_A \times {N}_B}}{{{N}_E}}} \right) \end{eqnarray*}

Similarly, the concentration of each individual target (λ_A_, λ_B_), irrespective of linkage, can be calculated as:


(5a) \begin{eqnarray*} {{\rm{\lambda }}}_A = \ln \left( {{N}_{tot}} \right)\ - {\rm{ln}}\left( {{N}_{notA}} \right) \end{eqnarray*}

(5b) \begin{eqnarray*} {{\rm{\lambda }}}_B = \ln \left( {{N}_{tot}} \right)\ - {\rm{ln}}\left( {{N}_{notB}} \right) \end{eqnarray*}

For each individual target the percentage of linked targets among the total concentration can now be calculated as:


(6a) \begin{eqnarray*} \% {A}_{linked} = \frac{{{{\rm{\lambda }}}_{\underline {AB} }}}{{{{\rm{\lambda }}}_A}}\ \ \times 100 \end{eqnarray*}

(6b) \begin{eqnarray*} \% {B}_{linked} = \frac{{{{\rm{\lambda }}}_{\underline {AB} }}}{{{{\rm{\lambda }}}_B}}\ \ \times 100 \end{eqnarray*}

### Proof of concept

To prove that the percentage of linked targets can be correctly estimated using the methodology described above, different voluminal ratios of the linked and unlinked target solutions were mixed at volumes (V) of linked targets at 100%, 90%, 75%, 50%, 25%, 10%, 5% and 0%. From the 100% and 0% mixtures the absolute concentration (c) of each gene in the linked and the unlinked samples were determined by ddPCR according to the protocol above. These are then used to calculate the theoretical ratio of linked to unlinked target genes:


(7) \begin{eqnarray*} \% targe{t}_{linked} = \frac{{{V}_{linked} \times {c}_{linked}}}{{{V}_{linked} \times {c}_{linked} + {V}_{unlinked} \times {c}_{unlinked}}}\ \times 100 \end{eqnarray*}

The validity of linkage detection for each gene was then assessed by comparing this theoretical linkage percentage with the value determined for each of the mixtures using the statistical evaluation of the duplex ddPCR assay.

### Environmental samples

Environmental wastewater samples of the influent and effluent of the wastewater treatment plant in Dresden-Kaditz Germany (51.072 N, 13.678E) were collected in sterile flasks, and stored at 4ºC until DNA extraction for a maximum of 12 h. Three replicate grab samples of 1 L each were taken for the influent and effluent on 24.03.2022. From each of the samples biomass was collected by filtration from a total volume of 130 ml for influent and 800 mL for effluent samples. Total environmental DNA was extracted from the filters using the DNeasy PowerWater kit (Qiagen) following the manufacturer's instructions. DNA concentration and purity were estimated spectrophotometrically. All DNA was stored at −20°C until use for ddPCR analysis.

### DNA shearing

**Figure 2.**
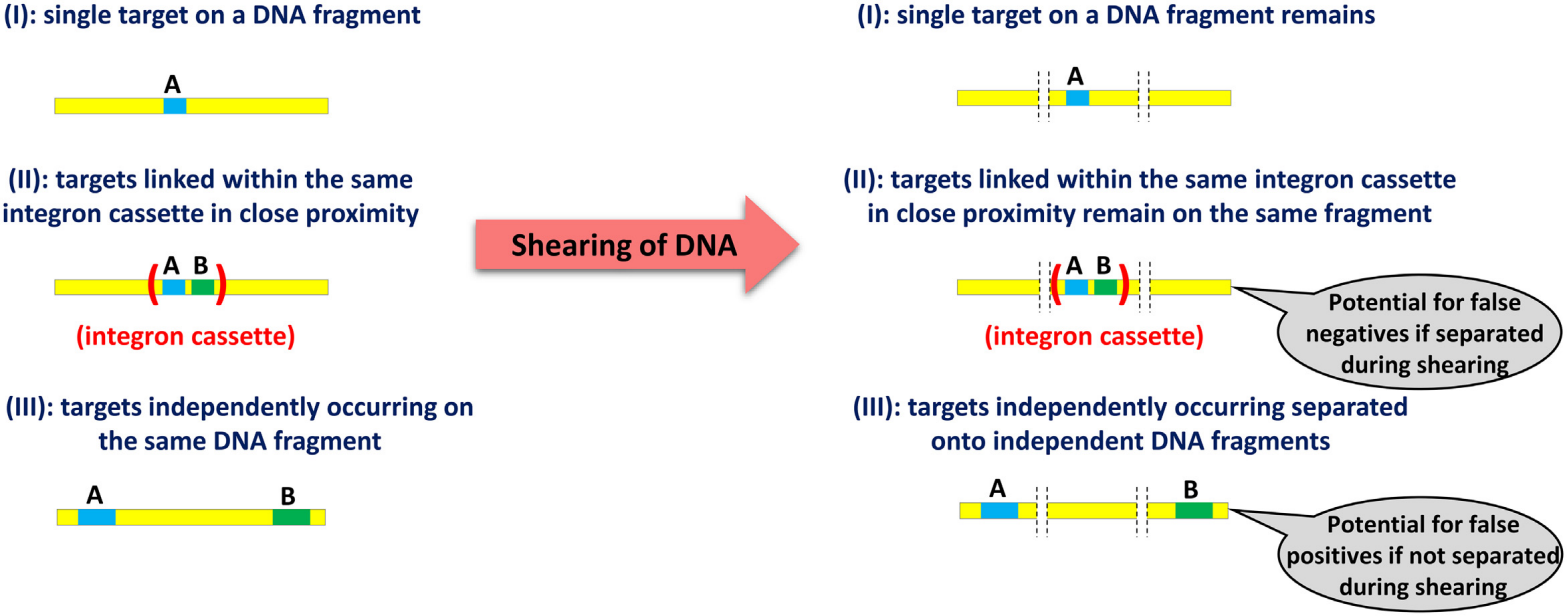
Shearing of DNA into fragments of a specific sizes allows distinguishing between both targets (**A** and **B**) occurring on one and the same DNA fragment due to either linkage within an integron cassette or both genes appearing independently. Fragmentation size allows adjusting the rate of false positive and false negative detection of linkage.

The two target genes can appear on one and the same DNA fragment, such as a bacterial chromosome, either linked in an individual integron cassette or independent of one another at random locations. To allow reliable analysis of their linkage within the same integron cassette, the DNA needs to be sheared into fragments of a specific size ahead of the analysis (Fig. 2).

The size of the fragments generated during the process of DNA shearing crucially affects the probabilities of false positive (*P*_FP_) and false negative outcomes (*P*_FN_). As a false positive outcome we regard cases where the two different target genes are detected on one and the same DNA fragment even though the targets are not linked (e.g. they are not part of a common mobile gene cassette). Conversely, false negative refers to the failure of detecting actual linkage between the targets (e.g. by being embedded on a common gene cassette) in response to the DNA fragmentation during the shearing process (Fig. 2).

As long as the target sequence is short in relation to the fragment length (L_F_), *P*_FP_ can be approximated as the probability of jointly detecting the two unlinked targets in any DNA fragment obtained upon shearing of a genome. Thus, if the total length of the genome is denoted L_G_, *P*_FP_ can be approximated by Eq. 8.


(8) \begin{eqnarray*} {P}_{FP} = \frac{{{{\rm{L}}}_F}}{{{{\rm{L}}}_G}}\ \end{eqnarray*}

The probability of false negative results is not only a function of L_F_, but it also depends on the length of the hypothetical gene cassette (L_C_) harboring the two targets and the actual position of the targets within the cassette. If we focus on cases where L_C_ < L_F_, i.e. a cassette can be sheared into no more than two fragments and the two targets are positioned in maximum distance within the cassette (representing the worst case scenario), *P*_FN_ can be approximated by Eq. 9.


(9) \begin{eqnarray*} {P}_{FN} = \frac{{{{\rm{L}}}_C}}{{{{\rm{L}}}_F}}\ \end{eqnarray*}

Worst-case estimates of *P*_FP_ and *P*_FN_ are reported for a range of assumed fragment lengths (Table 2). For these simulations, based on the literature, we assumed an average bacterial chromosome size L_G_ of 5 Mbp (Land et al. 2015) and the maximal length of clinically relevant Class 1 integron cassettes for L_C_ of 5 kbp (Partridge et al. 2009). This provides insights into the worst case scenario of the genes being linked but occurring in the same cassette at a maximal distance of 5 kbp. Since the two types of error show opposing dependencies on the fragment length, DNA shearing needs to target at intermediate values of L_F_ such that *P*_FP_ and *P*_FN_ are in balance. Under the given scenario, reasonable target values of L_F_ are in the range of 20–50 kbp.

**Table 2.** Probabilities of false positive (*P*_FP_, Eq. 8) and false negative outcomes (*P*_FN_, Eq. 9) in relation to assumed fragment lengths (L_F_) resulting from DNA shearing. Reported numbers refer to a scenario where L_G_ = 5 Mbp (total length of genome) and L_C_ = 5 kbp (length of gene cassette). Note that the values reported for P_FN_ represent worst-case estimates.

L_F_ (kbp)	*P*_FP_	*P*_FN_
10	0.002	0.5
20	0.004	0.25
50	0.01	0.1
100	0.02	0.05

Accordingly, a fragment length of 20 kbp was chosen for this proof of concept study to achieve a rate of false positive detection of less than 0.4% of the cases. The Covaris® g-TUBE (Covaris, Woburn, MA, USA) was used to shear the genomic DNA into the selected fragment sizes of around 20 kb, according to the protocol provided by the manufacturer. Success of the shearing process into fragments of the desired length was confirmed using gel electrophoresis.

## Results

### Reliable detection of different levels of linkage between the two target genes *sul1* and *intI1* from model samples

To determine if linkage between *sul1* and *intI1* is detectable using the statistical evaluation of the proposed multiplexed ddPCR method, initially the plasmid on which the two targets are linked and the unlinked PCR products of the two targets were used as templates. In the linked control sample, the plasmid DNA molecule hosting the linked copies of the two target genes was added at approximately 1500 copies µL^−1^. The concentrations of the two target genes measured using the multiplexed ddPCR protocol was 1510.0 ± 26.9 (mean ± SD) copies µL^−1^ for *sul1* and 1448.0 ± 20.0 copies µL^−1^ for *intI1*. This accounted for an acceptable average detection efficiency of 100.7% and 96.5% and further confirmed that both genes indeed appeared in a single copy on the used model plasmid.

**Figure 3.**
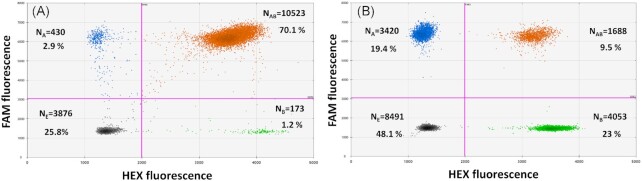
Data output from ddPCR experiments using the 100% (**A**) and 0% (**B**) linkage controls. N is the number of accepted droplets analyzed. Grey: Empty droplets (N_E_), negative for both targets; Blue: droplets positive only for target A, here *sul1* (N_A_); Green: droplets positive only for target B, here *intI1* (N_B_); Orange: droplets double-positive for both targets including those due to physical linkage and due to chance (N_AB_).

Obtained linkage percentages were calculated from the distribution of droplets (Fig. 3A) after correcting for stochastic effects (see M&M for calculation) as 92.42 ± 0.62% (*sul1*) and 96.34 ± 0.19% (*intI1*). The significant difference from the expected linkage percentage of 100% (both *P*<0.05, two-tailed *t*-test, dF = 5), can be explained by the above mentioned difference in detection efficiencies of the two target genes or due to separation of the two targets based on shearing through e.g. pipetting of the linked control plasmid, which ultimately results in a limited number of single positive droplets. The rate of false negatives, and hence the average underestimation of gene linkage of 7.58% and 3.66% for the two genes (rate of false negatives) falls still far below the worst case scenario calculated for the specific shearing fragment length of a maximal 25% (Table 2).

In the unlinked control, we still expect to see double-positive droplets due to chance in the ddPCR partitioning process. Here, 9.5% ± 0.13% of droplets that contained both targets were obtained in experiments with unlinked targets (Fig. 3B, orange cluster). Statistical evaluation of the results revealed, however, that these occurred indeed exclusively by chance with the obtained linkage percentages for the two target genes calculated as 0.31% ± 1.05% (*sul1*) and 0.26% ± 0.92% (*intI1*) (see M&M for calculation). These results were not significantly different from the expected linkage of 0% for either of the genes (*p_sul1_*= 0.658, *t_sul1_*= 0.515; *p_intI1_*= 0.663, *t_intI1_*= 0.506; two-tailed *t*-test, dF = 5), hence demonstrating that if targets are not linked, no false positives for their potential association on the same DNA molecule can be detected.

**Figure 4.**
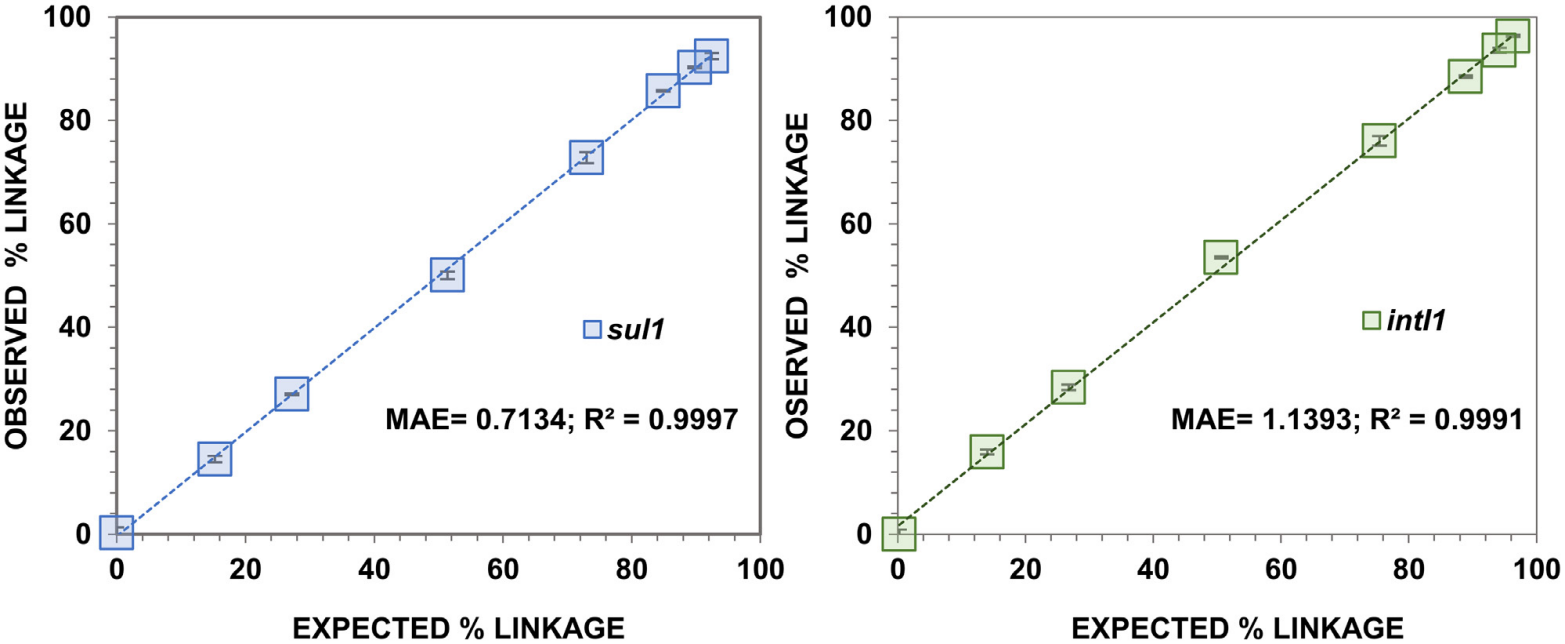
Linear correlation between theoretical and experimentally calculated linkage percentage for the two target genes *sul1* (**A**) and *intI1* (**B**). Tested samples were prepared at eight defined volumetric mixtures of linked and unliked targets and measured with three technical replicates by multiplexed ddPCR (n = 24). MAE, mean absolute error in percentage (%).

When validating the method by using mixtures of linked and unlinked targets at defined linkage percentages the experimentally obtained values were very well correlated with the theoretically expected ones based on the correlation coefficient and the mean absolute error for the linkage of both target genes, *sul1* (R^2^ = 0.9997, MAE = 0.71%, n = 24) and *intI1* (R^2^ = 0.9991, MAE = 1.14%, n = 24) (Fig. 4). Further the method proved robust as the standard deviation of the technical replicate measurements at different linkage ratios was consistently low (0.18%–1.08%).

Consequently, the proposed method provides reliable values for the linkage between the target genes, with the level of false positive detection of linkage being negligible and the level of false negative detection of linkage at high linkage percentages being within the level expected due to methodological constraints based on the optimization of the shearing procedure. This allowed applying the method to more complex environmental samples to detect the linkage of the *sul1* gene with Class 1 integrons.

### Abundance of target genes in wastewater influent and effluent samples

**Figure 5.**
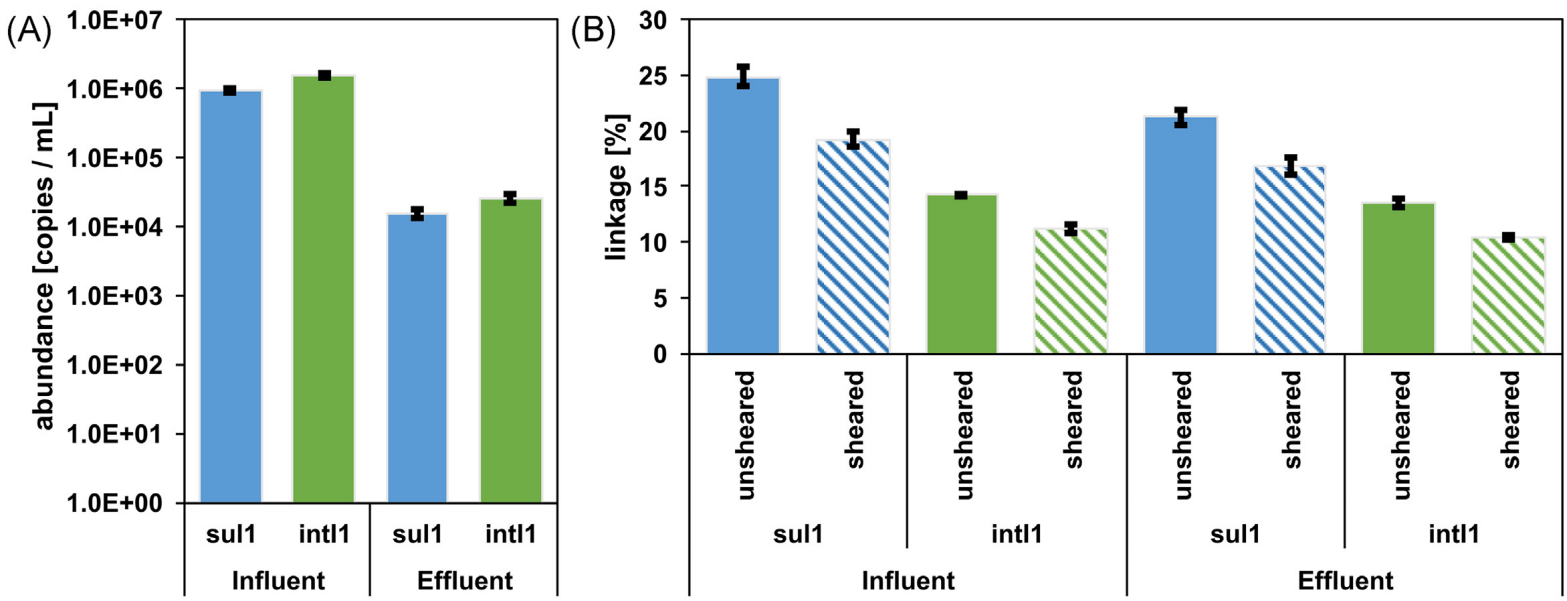
Abundance (**A**) and linkage (**B**) of the two target genes *sul1* and *intI1* in wastewater influent and effluent samples. Linkage was determined for unsheared as well as sheared DNA (average fragment size = 20 kbp).

The optimized protocol was applied to DNA extracted from environmental water samples obtained from the influent and effluent of a wastewater treatment plant to first determine the abundance of the target genes. The abundance of both target genes *sul1* and *intI1* was significantly higher in the influent compared to the effluent of the wastewater treatment plant (*p_sul1_*<0.0001, *t_sul1_*= 36.72; *p_intI1_*<0.0001, t*_intI1_*= 41.18; dF = 10, two-tailed *t*-test). In the influent the abundance of *sul1* was determined as 9.15 ± 0.60 × 10^5^ copies mL^−1^ and the abundance of *intI1* was 1.58 ± 0.09 × 10^6^ copies mL^−1^, while in the effluent these numbers were reduced by 58-fold to 1.56 ± 0.22 × 10^4^ copies mL^−1^ (*sul1*) and 64-fold to 2.49 ± 0.34 × 10^4^ copies mL^−1^ (*intI1*) (Fig. 5A).

### Linkage between the *sul1* and *intI1* target genes in unsheared DNA of the environmental samples

Based on the statistical evaluation of the multiplexed ddPCR assay, the detected initial linkage when applying the unsheared DNA, was also reduced when comparing the influent with the effluent. For *sul1* the percentage of detected gene copies that were linked to *intI1* was 24.83 ± 0.88% in the influent and significantly reduced to 21.25% ± 0.71% in the effluent (*P* = 0.0054, *t* = 5.47, dF = 4, *t-*test, Fig. 5B). Equally for *intI1* the percentage of gene copies linked to *sul1* was significantly lowered during passage through the wastewater treatment plant from 14.21 ± 0.17 to 13.53% ± 0.30% (*P* = 0.0278, *t* = 3.38, dF = 4, *t*-test, Fig. 5B). The significantly lower linkage of *intI1* compared to *sul1* in all samples is here due to the significantly higher abundance of *intI1* in both sample types. However, the unsheared DNA does not account for the co-abundance of the two genes on longer DNA fragments irrespective of co-occurrence within the context of a Class 1 integron cassette (Fig. 2).

### Shearing of DNA reveals the true linkage between the *sul1* and *intI1* target genes in environmental samples

DNA was subsequently sheared to the optimal fragment size of ∼20 kbp. Shearing did not have an effect on the detection of either of the two target genes as the ratio of *sul1* to *intI1* between the unsheared (Influent: 0.57 ± 0.02; Effluent: 0.64 ± 0.01) and sheared assays (Influent: 0.58 ± 0.00; Effluent: 0.61 ± 0.02) remained insignificantly affected (*P*_Inf_ = 0.4588, *t*_Inf_ = 0.819; *P*_Eff_ = 0.1516, *t*_Eff_ = 1.769; dF = 4, two-tailed *t*-test). However, shearing had a significant effect in reducing the detected linkages of each of the genes for each sample type (Fig. 5B; all *P* = 0.0001–0.0017, dF = 4, two-tailed *t*-test). For *sul1* the linkage to *intI1* after shearing was reduced to 19.25% ± 0.62% in the influent and 16.87 ± 0.73% in the effluent. For *intI1* the linkage after shearing was determined as 11.24% ± 0.43% in the influent and 10.36% ± 0.18% in the effluent.

Taking these new linkage values into account, the wastewater treatment process did not only reduce the gene abundance of the two target genes, but also significantly reduced their linkage (*P* = 0.0125, *t* = 4.31, dF = 4, two-tailed *t*-test) (Fig. 5B). A lower linkage of *sul1* with Class 1 integron cassettes indicates a lower potential of mobilization for this ARG as it appears less frequently in the vicinity of the MGE. However, the reduction in linkage of ∼14% was only very slight compared to the 10- to 100-fold reduction in gene absolute abundance.

Overall, this demonstrates that the method can easily be applied for linkage analysis of ARGs with MGEs within environmental samples. Based on the results obtained without DNA shearing, the linkage between the two genes would have been overestimated by around 20% for each of the genes in each of the samples, due to independent occurrence of both targets (outside of the identical integron cassette) on the same DNA fragment. Thus, DNA fragmentation is an absolute necessity for an accurate detection of ARG linkage with integron cassettes that can appear chromosomally.

## Discussion

We here present a novel, multiplexed ddPCR based approach to simultaneously assess the abundance of individual target genes and their linkage from complex environmental samples. This allows estimating the linkage of ARGs to specific MGEs, hence their potential to be mobile within a given sample. Such an assessment of the mobility potential is an important contributor to the risk an environmental ARG is posing towards human health as it describes the likelihood of an ARG being able to spread to a human pathogen (Zhang et al. 2021). This likelihood of ARG mobilization is however not guaranteed, but far increased when an ARG occurs in close proximity with an MGE indicator gene. The determination of this mobility potential is exemplarily shown for the sulfonamide resistance gene *sul1* and its association with Class 1 integron cassettes based on the integron integrase gene *intI1*. Using model DNA target molecules with either each gene in isolation or both genes linked, we demonstrate that the theoretical linkage in mixtures of these targets can indeed be accurately quantified. Further, the applicability to assess such linkages in environmental samples is demonstrated based on wastewater influent and effluent samples.

For integron cassettes that can equally be part of chromosomes or MGEs (Zhang et al. 2018) we, through means of statistical modeling, demonstrate that the rates of false positive and false negative detections depend directly on the shearing fragment length of the sample DNA. In this proof of principle study, a conservative fragment length of 20 kbp for Class 1 integron cassettes of a maximal size of 5 kbp (Partridge et al. 2009) was chosen to keep the rates of false positives below 0.4% based on the mathematical assessment of the worst case scenario of both genes being on opposite sides of the integron cassette. This cutoff comes with a tradeoff of a relatively high 25% theoretical maximal false negative detection rate. In this context it is important to point out that while positive association with the tested MGE is an indicator of potential mobility, negative association does not imply no mobility, as the ARG in question could be associated with a different MGE than the one tested. As positive association has hence a higher degree of meaning, optimizing the method for a low rate of false positives rather than a low rate of false negatives became imperative. Furthermore, using our reference plasmid with linked targets in close proximity the experimentally detected rate of false negative detection of linkage was far below the theoretical maximum and ranged between 3% and 8%, hence indicating that optimizing fragment length for low rates of false positive detection should indeed be favored.

We further prove that DNA fragmentation is an absolute necessity for an accurate detection of ARG linkage with integron casettes in environmental samples as the linkage between the two genes in the wastewater samples is overestimated by around 20% in the absence of the fragmentation process. Obviously when applying this method in the future, different fragment sizes for different combinations of targets lead to different tradeoffs between false positive and false negative detection and need to be carefully decided based on the task at hand.

Different approaches or combinations of molecular and culture-based methods have been used to link ARGs to MGEs in non-clinical environments (Rice et al. 2020): One of the most commonly applied methods is metagenomic assembly of genomes or contigs (Liu et al. 2019, Dai et al. 2022, Kneis et al. 2022). However, for this approach assembled contigs have to be long enough to simultaneously contain ARGs as well as the genetic indicators of the MGEs in question. Due to sequencing depth constraints, only the most common ARGs with a relative abundance of more than 10^−4^ copies per 16S copy can be detected (Gweon et al. 2019). However, the relative abundance of ARGs and MGEs in most natural environments is low, which increases the requirements regarding sequencing depth and hence the costs (Klümper et al. 2022).

The most promising molecular approach currently available to assess ARG mobility is Inverse PCR of the ARG in question combined with long read sequencing of the amplified surrounding regions to identify clusters in which ARG are co-occurring with genes associated with MGEs (Pärnänen et al. 2016). Still this approach is also relying on sequencing. Furthermore, due to the PCR amplification steps involved, it does not allow for a quantitative analysis of mobility. If such associations with MGEs are common or rare, remains however crucial information needed in risk assessment. However, PCR based approaches, including Inverse PCR and the here presented ddPCR approach, have several orders of magnitude improved detection limits compared to metagenomics (Link-Lenczowska et al. 2018) making them also suitable for those ARGs that are rare in a given environment. For example, in the case of the here presented ddPCR method the detection limit is as low as 3 linked target copies per ddPCR reaction, which is derived from resolving the governing Poisson distribution as the minimum number of positive droplets needed to detect linkage with 95% confidence. Moreover, the limit of quantification was determined similarly as 9 linked target copies per reaction by considering the confidence limit of the relative error. Specifically, if the observed number of linked target copies per reaction (x) is ≥ 9, the observed linkage percentage falls within the interval x ± 100% with a probability of 95% or higher. Despite this improved detection limit, PCR based approaches have the disadvantage of needing specific primers for each individual specific target. Consequently, novel ARGs or those ARGs not expected in a certain sample might be missed as they might not be tested for (Klümper et al. 2022).

A major disadvantage of the above discussed molecular approaches, including the here presented method, is that mobility is inferred from genetic information. For example, ARGs encoded within a Class 1 integron casette are only transferable between bacterial cells if this mobile Class 1 integron is localized on a transferable plasmid, conjugative ICE or a transposon (Gillings et al. 2008). This limitation can exclusively be overcome by culture-based methods such as exogenously capturing MGEs through bi- or triparental matings from non-cultivated donor cells into defined recipient cells (Heuer et al. 2012, Shintani et al. 2020). Unlike the molecular approaches, here the phenotypic resistance as well as transferability, and the factual mobility of ARGs can be demonstrated. However, due to the cultivation steps these methods are rather labor intensive and slow. Furthermore, only a minor subset of plasmids within the given sample that can efficiently transfer to the recipient strain can be captured, hence not allowing for a quantitative assessment of the linkage of ARGs and MGEs. Yet, a combination of this quantitative ddPCR method with cultivation-based approaches to confirm transferability and phenotypes could provide significant ecological insights into the nature of mobile ARGs.

Despite their individual weaknesses, the existing methods can provide promising insights into ARG mobility potential in the environment. Still, the above mentioned alternative approaches are either technologically complicated and rely on sequencing analysis which is expensive, time consuming, and requires specialized bioinformatics knowledge for data analysis, or are mainly qualitative or semi-quantitative at best, which makes risk assessments based on ARG linkage with MGEs difficult.

A major advantage of the here presented ddPCR approach lies in the cheap, rapid and straightforward generation of quantitative results. While sequencing and subsequent sequence analysis take time, computing power and expert bioinformatics knowledge, the ddPCR approach allows the generation of results within hours and the evaluation can be automated using simple scripts for statistical analysis. In addition, the presented method has a high potential to be up-scaled to multiple targets. Currently, ddPCR machines are able to detect up to two fluorochromes simultaneously, with future developments expected to increase this number. Furthermore, it can straightforwardly be extended to other ARGs or genes of interests and different groups of MGEs. The only necessary means to achieve this is the design of appropriate sets of primers and probes that allow multiplexed ddPCR analysis for the respective targets. For example, the association of a given ARG with different groups of plasmids in an environmental sample can easily be tested. In that case the shearing process could even be omitted, as plasmids already provide separate DNA entities from chromosomes. Hence the rate of false positives would be dramatically reduced, which allows for the detection of ARG-plasmid associations directly from extracted environmental DNA. Here the presented method would benefit from a combination with Inverse PCR with long read sequencing (Pärnänen et al. 2016) which allows determining the most interesting ARG-MGE associations, which could subsequently be quantified using the multiplexed ddPCR approach with targeted primers. Different degenerate primers for different groups of plasmids even already exist from replicon- or MOB-typing of plasmids (Alvarado et al. 2012, Garcillán-Barcia et al. 2015, Villa and Carattoli 2020) and could be adapted for this approach. Detection of ARG-plasmid associations could hence be carried out for individual plasmid groups using different fluorophores. In addition the use of already existing degenerate primers (Garcillán-Barcia et al. 2015) or mixtures of primers targeting multiple plasmid groups that all use the identical fluorophores could provide a general percentage of ARG-plasmid linkage in a given sample. Such approaches could be especially valuable considering the possibility of combining them with microfluidic enrichment of ddPCR droplets based on positive fluorescence signals for either targeted sequencing of those plasmids that do contain environmentally relevant ARGs (Eastburn et al. 2015) or identification of MGE hosts by sequencing of the 16S rRNA encapsulated in single cell ddPCR droplets that score positive for the respective ARG. Such, currently lacking, MGE-host associations in a given environment would be a further step towards successful risk assessment endeavors.

In summary, the presented, multiplexed ddPCR approach proves to be a suitable and rapid, and cost-efficient approach to simultaneously assess the abundance of individual target genes and their linkage. With a straightforward protocol, high adaptability towards new targets and potential for extension through additional methods, it is hence a promising method for the assessment of ARG mobility. The suitability of ddPCR applications in environmental surveillance protocols has already been demonstrated as it has recently gained traction as a cost-efficient method included in global wastewater monitoring initiatives of the COVID-19 pandemic (Alygizakis et al. 2021, Dumke et al. 2021, Pillay et al. 2021). With ARG mobility as one of the main determinants for ARG risks towards human health (Zhang et al. 2021), this targeted approach might hence be a suitable addition to be considered for future, global environmental AMR monitoring frameworks to assess ARG mobility.
